# BTR: a bioinformatics tool recommendation system

**DOI:** 10.1093/bioinformatics/btae275

**Published:** 2024-04-25

**Authors:** Ryan Green, Xufeng Qu, Jinze Liu, Tingting Yu

**Affiliations:** Department of Computer Science, University of Cincinnati, Cincinnati 45219, United States; Department of Biostatistics, Virginia Commonwealth University, Richmond 23284, United States; Department of Biostatistics, Virginia Commonwealth University, Richmond 23284, United States; School of Computing, University of Connecticut, Storrs 06269, United States

## Abstract

**Motivation:**

The rapid expansion of Bioinformatics research has led to a proliferation of computational tools for scientific analysis pipelines. However, constructing these pipelines is a demanding task, requiring extensive domain knowledge and careful consideration. As the Bioinformatics landscape evolves, researchers, both novice and expert, may feel overwhelmed in unfamiliar fields, potentially leading to the selection of unsuitable tools during workflow development.

**Results:**

In this article, we introduce the Bioinformatics Tool Recommendation system (BTR), a deep learning model designed to recommend suitable tools for a given workflow-in-progress. BTR leverages recent advances in graph neural network technology, representing the workflow as a graph to capture essential context. Natural language processing techniques enhance tool recommendations by analyzing associated tool descriptions. Experiments demonstrate that BTR outperforms the existing Galaxy tool recommendation system, showcasing its potential to streamline scientific workflow construction.

**Availability and implementation:**

The Python source code is available at https://github.com/ryangreenj/bioinformatics_tool_recommendation.

## 1 Introduction

Bioinformatics researchers use computational components to analyze and interpret large biological data. The practice of creating reproducible, scalable, and shareable analysis pipelines (Wratten *et al.* 2021) has gained prominence. Over the years, various systems and standards, including NextFlow (Di Tommaso *et al.* 2017), Common Workflow Language (Crusoe *et al.* 2022), Snakemake (Mölder *et al.* 2021), and Galaxy (Afgan *et al.* 2018), have emerged to streamline workflow creation. These systems aim to simplify the process for individuals lacking technical expertise, offering features like accessing a shared toolbox, optimizing resource use, handling installations, and resolving versioning issues through pre-configured virtual environments like Anaconda (Anaconda Software Distribution 2016) and Docker (Merkel 2014).

Developing new workflows is a challenging task, requiring a thorough understanding of tools in the specific domain and their interactions at different stages. Experienced bioinformaticians may possess the domain knowledge and coding expertise needed for pipeline compositions. In contrast, newer researchers, particularly those with limited computational backgrounds, may rely more on finding and using existing tools. Understanding available tools, their functionalities, and integration possibilities is crucial. Obtaining this information can be time-consuming, involving hours of web-surfing with abstract search concepts to find a desired function. The rapid growth of Bioinformatics has expanded the tool catalog, complicating selection. For instance, Galaxy hosts over 9200 tools, with an average workflow comprising 13 tool steps. Galaxy Toolbox experienced a 53% growth from 2016 to 2018 (Afgan *et al.* 2018), demonstrating roughly 40%–50% growth every two years. The influx of new information complicates the reuse of functions and hinders the discovery and integration of new tools into workflows, exacerbated by a lack of training material.

It is impractical for human researchers to be fully versed in the complete tool catalog. Automated solutions aid in tool selection, with methods like EDAM (Ison *et al.* 2013) and bio.tools (Ison *et al.* 2015), a community-sourced platform with over 28 000 tools. However, querying alone faces challenges, such as identifying the most suitable tool compatible with an existing workflow. The Automated Pipeline Explorer (APE) (Kasalica and Lamprecht 2020) generates abstract workflow possibilities but requires manual validation. Workflow INstance Generation and Selection (WINGS) (Gil *et al.* 2011a, 2011b) automatically finds implementations but needs expertise in high-level workflow construction. The Galaxy tool recommender (GTR) system by Kumar *et al.* (2021) suggests downstream compatible tools but lacks specificity due to vast compatibility sets. Previous methods often focus on specific implementations or defining workflows abstractly. An ideal system would perform both in one step. Moreover, methods using information from a workflow-in-progress often consider only a single step or linear sequence, not fully representing the branching and winding workflow structures in practice.

In this article, we aim to address the on-demand Bioinformatics tool recommendation problem during workflow realization and construction by introducing the *B*ioinformatics *T*ool *R*ecommendation system (BTR). We model workflow construction as a session-based recommendation (Li *et al.* 2017, Wu *et al.* 2019, Ma *et al.* 2020) problem and leverage emerging graph neural network technologies (Scarselli *et al.* 2009, Li *et al.* 2016) to enable a workflow graph representation capturing extensive structural context. This approach represents the workflow as a directed graph, with a variant of the system constrained to employ linear sequence representations for comparison with other methods.

We conduct a comprehensive evaluation of BTR and its variants with two extensive Galaxy databases, each comprising over 1250+ unique tools and a combined 7000+ workflows. Additionally, we compare BTR to a baseline method designed to solve a similar Bioinformatics tool recommendation problem. Lastly, we explore the viability of BTR in the age of large language models. It is found that BTR demonstrates considerable performance in the direct tool recommendation problem, significantly outperforming the baseline system. Furthermore, large language models do not surpass the performance of our specialized system and show degraded performance given the same inputs and task.

## 2 Materials and methods

### 2.1 Preliminary definitions and representations

To accurately describe the deep learning steps of our proposed tool recommendation system—BTR, we first define how individual tools and workflows are represented. BTR requires a toolbox of bioinformatics tools, the building blocks of potential workflows. Thus, a “tool” is at the lowest level of abstraction obtained from a workflow. Bioinformatics libraries often contain functions, each considered a tool in our toolbox. For instance, the BEDTools library (Quinlan and Hall 2010) comprises essential functions like *annotate* and *map*, each representing a self-contained task. In this case, functions like *bedtools_annotate* and *bedtools_map*, along with their descriptions, are added as tools to *T*. Formally, tools are represented by a 〈toolbox ID, description〉 pair.

A workflow defines the execution sequence alongside input and output connections between bioinformatics tools to perform specific tasks. Workflows are represented following the Abstract Workflow Representation (AWR) format, akin to graphical displays within workflow management systems (Mölder *et al.* 2021, The Galaxy Community 2022). The AWR W=(S,C) comprises a list of steps *S* (|S| ≥ 1, nodes of the graph) and connections *C* between those steps (edges). Each step s∈S points to a tool of *T*. Multiple invocations of a tool throughout a workflow are allowed, and duplicates are permitted. The connections *C* represent the data flow between tools throughout the workflow, with workflows represented by the AWR exhibiting properties of directed acyclic graphs (DAGs).

### 2.2 Problem description

**Figure 1.**
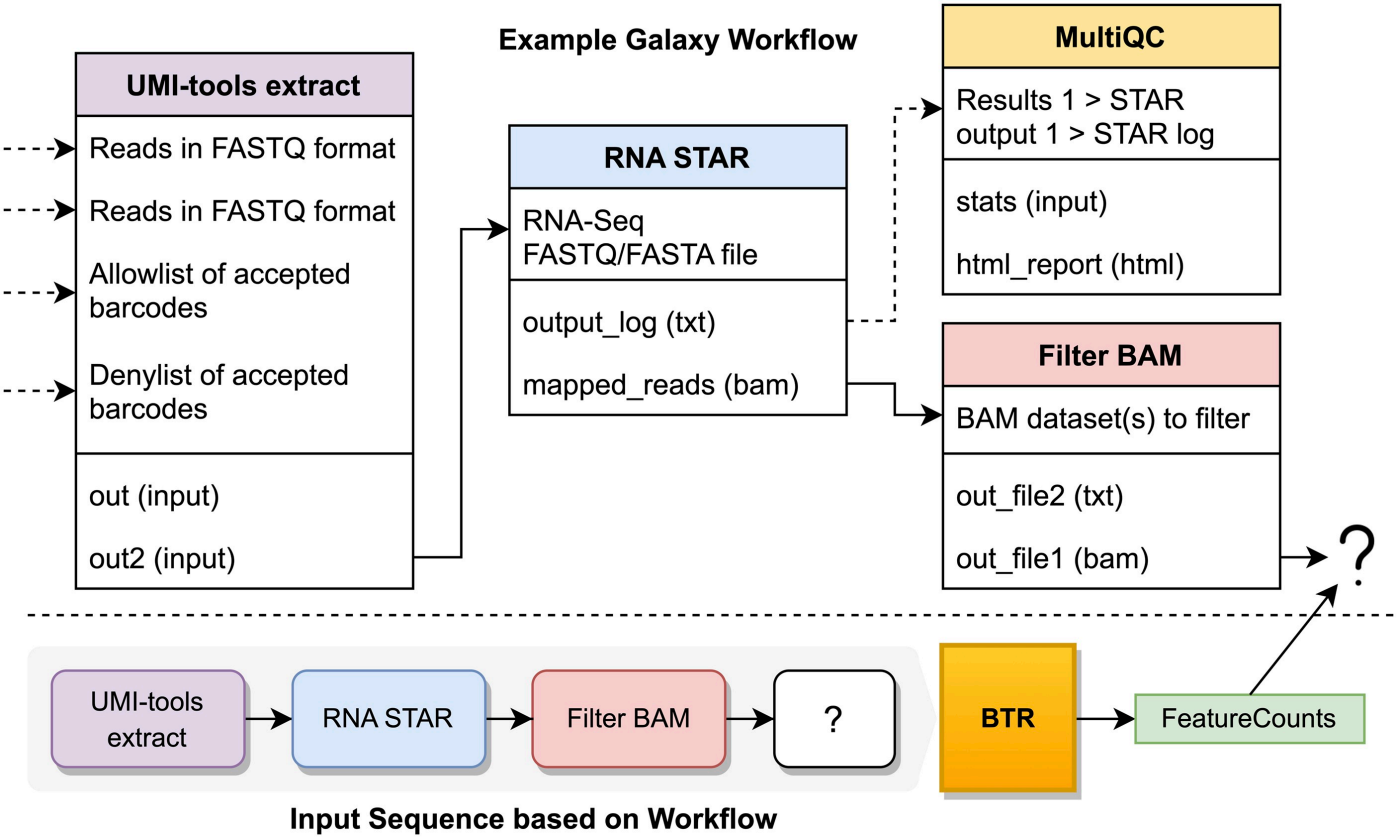
A showcase of tool recommendation by BTR. The workflow-in-progress (top) is from a tutorial^1^ about Single-Cell data pre-processing. The figure mimics how tool nodes appear within the Galaxy editor, and the connections between their inputs/outputs. In this case the user desires a tool to follow the out_file1 (bam) of Filter BAM. The next functionality from the tutorial is to create a count matrix using UMI-tools count, but this tool is dependent on a call to FeatureCounts to annotate the BAM reads with gene name. The bottom portion of the figure shows the abstraction of workflow graph/sequence to capture only tool identities and their interconnections, which serves as the input for BTR. Note that only the upstream dependencies of the tool at the desired position (denoted by “?”) are included. BTR correctly outputs FeatureCounts as the highest-ranked tool from 1250+ choices.^1^https://training.galaxyproject.org/training-material/topics/single-cell/tutorials/scrna-preprocessing/tutorial.html.

In the context of machine learning, we frame the tool recommendation task as a regression problem predicting the most likely tool for any given workflow-in-progress. Probabilities are calculated for each candidate in the toolbox *T*. The input query to BTR is an AWR of the incomplete workflow up to the desired point of recommendation, referred to as the prefix-AWR. Two architecture variants are discussed in this article, ${\text{BTR}}^{g}$, which operates on full workflow graph representations, and ${\text{BTR}}^{s}$, that consumes linear tool sequences. The output of BTR is a recommended tool that can occur in the prefix-AWR after a user-defined list of preceding steps *R*. BTR assumes that output data from steps of *R* will directly feed into the recommended tool. The system produces a set of probabilities *P* for all tools in *T* from which it can choose optimal candidates. In the example of BTR from Fig. 1, T = [“UMI-tools extract,” “RNA STAR,” “Filter BAM,” “MultiQC,” “FeatureCounts,” …], steps *S* = [0, 1, 2], connections *C* = [(0, 1), (1, 2)]. BTR can be considered a function P=BTR(w=(S,C),R=[2],T=T). This yields a possible recommendation of T[argmax(P)]= “FeatureCounts.”

### 2.3 Workflow-in-progress as graph and sequence query

${\text{BTR}}^{g}$ is variant of the BTR architecture that employs a graph representation of a workflow-in-progress, defined as a set of upstream nodes and their connections that a recommended tool will depend on. The model internally inserts a blank query node *q*, displayed as the “?” in Fig. 1, and creates directed edges from user-defined preceding steps *R* to the query node. The objective is to solve the tool that replaces *q*.

Harnessing the flexibility of BTR structure, we create the second variant ${\text{BTR}}^{s}$, where workflows are treated as linear sequences of ordered tool invocations instead of graphs. This variant is similar to the recently published approach of recommending Galaxy tools (Kumar *et al.* 2021). No query node is added to the input of ${\text{BTR}}^{s}$ because *R*, the list of preceding steps from Section 2.2, is automatically inferred to consist solely of the most recent step in the incomplete workflow sequence.

### 2.4 Tool recommendation using graph neural networks

**Figure 2.**
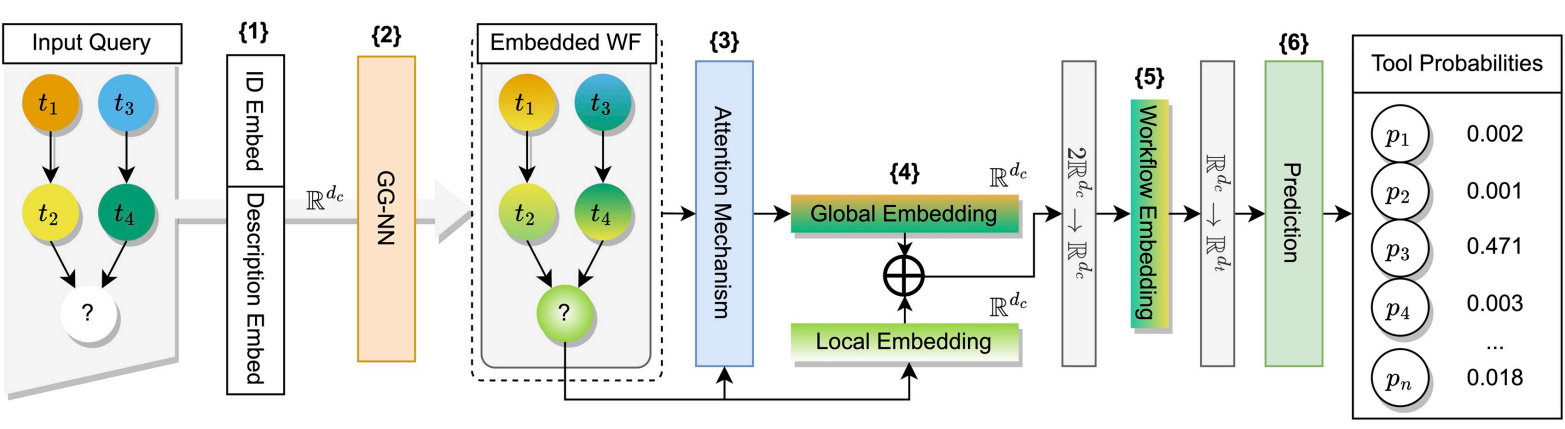
Overview of the BTR modeling architecture for Bioinformatics tool recommendation. BTR takes the input of Input Query in the format of a sequence or a graph. The prediction framework is composed of six major steps: {1} Each tool instance is encoded by an initial embedding layer (Section 2.4.1); {2} The initial embeddings continue to a Gated Graph Neural Network to learn contextural and structural features from neighboring nodes using full workflow graph; (Section 2.4.2); {3} An attention mechanism aggregates the latent graph node embeddings into a full workflow representation, which is concatenated with the representation of the last tool {4} and transformed to yield the final workflow representation vector {5} (Section 2.4.3), which is then compressed to the size of the tool embedding; {6} Tool probabilities are produced by similarity of compressed workflow representations to all tool embeddings from the toolbox (Section 2.5).

In this section the proposed deep learning model architecture as shown in Fig. 2 is described in detail.

#### 2.4.1 Tool embedding integrating NLP description

Embeddings of toolbox ID and corresponding semantic tool description are tied together as initial node features during graph learning. Natural language processing (NLP) techniques are applied to extract latent knowledge from the semantic tool description. Including such information as a node feature allows the model to gain access to a depiction of semantic tool similarity and thus improve correlation of the usage and relationships between tools.

Tool *description* is converted to a latent vector using a sentence encoder. Sentence encoders are language models that embed sentences into $R^{d_{e}}$ dimensional vectors that capture the semantic meaning of a sentence, useful for similarity calculation and transfer learning (Reimers and Gurevych 2019). For this task, we use PubMedBERT (Gu *et al.* 2021), a BERT (Devlin *et al.* 2019) model that is pre-trained from scratch on a large corpora of PubMed abstracts and full-text articles. PubMedBERT embeds sentences into 768-dimensional vectors, which becomes $d_{e}$. This encoder yields state-of-the-art results for many domain-specific NLP tasks and utilizes an in-domain vocabulary that allow s tokenization of many relevant biomedical terms. We use a version of PubMedBERT (https://huggingface.co/pritamdeka/PubMedBERT-mnli-snli-scinli-scitail-mednli-stsb) that is fine- for sentence embedding. This gives the representation $x_{e}\in R^{d_{e}}$.

The index of the tool in toolbox *T* is represented by a one-hot encoded vector $t_{\text{ind}}\in R^{u}$, where u=|T| is the total number of tools in the toolbox. This vector is multiplied with a matrix of learnable weights $W_{\text{tool}}\in R^{u\times d_{t}}$ yielding a latent vector $x_{t}\in R^{d_{t}}$, where $d_{t}$ is a hyperparameter representing the dimensionality of the tool ID vector. $W_{\text{tool}}$ is learned using back-propagation through time (Mozer 1995).

(1) $$x_{t}=t_{\text{ind}}\times W_{\text{tool}}.$$

To obtain the combined encoding $x\in R^{d_{t}+d_{e}}$, $x_{t}$, and $x_{e}$ are concatenated. The combined hidden dimension is referred to as $d_{c}=d_{t}+d_{e}$ throughout this section.

#### 2.4.2 Workflow learning using gated graph propagation

Next, workflow features and node relationships are extracted through the message passing and aggregation layer. This enables individual steps to capture both structural and feature information from their local neighbors. This capability is analogous (Hamilton 2020) to the behavior of convolutions in convolutional neural networks (Krizhevsky *et al.* 2017). Gated graph neural networks (GG-NNs) (Li *et al.* 2016) apply the intuitions of gated recurrent units (GRUs) (Cho *et al.* 2014) with the intention of better representing sequential data.

The methods of BTR are adapted for the tool recommendation problem based on the architecture of Session-based Recommendation with Graph Neural Networks (SR-GNNs) by Wu *et al.* (2019). Session-based recommendation is a technique of recommender systems that only considers the recent history or “current session” of the *user* when making predictions or recommending *items* (Li *et al.* 2017, Wu *et al.* 2019, Ma *et al.* 2020). The representation of *user* is based solely on in-session data and no historical or auxiliary information is included. Our work models workflow construction as a session-recommendation task where workflows are the “users” and tool steps are the “items.” This technique is applicable because the system should only consider the current workflow-in-progress. Wu *et al.* (2019) demonstrate how GG-NNs can be applied to the session-recommendation problem. They propose a model, SR-GNNs, that uses a GG-NN layer combined with attention mechanism (Vaswani *et al.* 2017) to perform next-item prediction. The model sees improved performance over several baseline algorithms on e-commerce data, some of which utilize recurrent models themselves (Hidasi *et al.* 2015, Li *et al.* 2017).

Let matrix $A\in R^{n\times 2n}$ define the adjacency between nodes of the graph, the horizontal concatenation of an $A^{\left(\text{out}\right)},A^{\left(\text{in}\right)}\in R^{n\times n}$, where $A_{i:}\in R^{n\times 2}$ are the two columns representing the out-directed and in-directed edges in A corresponding to a node $v_{i}\in V$. In this case of tool recommendation, edges are simply represented as **1** for present and **0** for nonexistent. During propagation of graph G=(V,E), the nodes $V=[v_{1},\ldots ,v_{n}]$ representations are updated by the following. Equation (2) performs message passing between the nodes of the graph using the outgoing and incoming edges defined in A. Here, $a_{i}^{\left(t\right)}\in R^{2n}$ is the extracted activation of node $v_{i}$. The remaining equations are those of GRU. Equations (3) and (4) are the update and reset gates, σ is the sigmoid function $\sigma (x)=1/(1+e^{-x})$. Equation (5) constructs a candidate state using the current state, reset gate, and previous state, where ⊙ is element-wise multiplication. Finally, Equation (6) uses the update gate to combine the previous state and candidate state to compute the final embedding. $W_{*}$, $U_{*}$ are learnable weight matrices and **b** is bias.

(2) $$a_{i}^{\left(t\right)}=A_{i:}^{⊺}{\left[v_{1}^{\left(t-1\right)},\ldots ,v_{n}^{\left(t-1\right)}\right]}^{⊺}+b.$$

(3) $$z_{i}^{\left(t\right)}=\sigma (W_{z}a_{i}^{\left(t\right)}+U_{z}v_{i}^{\left(t-1\right)}).$$

(4) $$r_{i}^{\left(t\right)}=\sigma (W_{r}a_{i}^{\left(t\right)}+U_{r}v_{i}^{\left(t-1\right)}).$$

(5) $$\tilde{v_{i}^{\left(t\right)}}=\text{tanh}(Wa_{v}^{\left(t\right)}+U(r_{v}^{\left(t\right)}⊙v_{i}^{\left(t-1\right)})).$$

(6) $$v_{i}^{\left(t\right)}=(1-z_{i}^{\left(t\right)})⊙v_{i}^{\left(t-1\right)}+z_{i}^{\left(t\right)}⊙\tilde{v_{i}^{\left(t\right)}}.$$

#### 2.4.3 Integrating local and global workflow embedding

Important contextual information is gained when looking at upstream tools in the workflow. To capture this, the intuition of Wu *et al.* (2019) is followed to model short-term and long-term preferences. A local and global workflow embedding, $w_{l},w_{g}\in R^{d_{c}}$, are computed and combined to aggregate the individual node embeddings into a full workflow representation. The local embedding is the representation of the most recent tool node in the workflow. We imagine there may exist rather general tools that can appear in a workflow and do not give useful contextual information; some relationships or tools in a workflow may not be as important as others. An attention mechanism (ATTN) is utilized to empower the model in discerning the significance of each tool in relation to others. The local workflow embedding is used as the latest hidden state in the attention mechanism. Given the set of *n* node embeddings in a workflow, $s\in R^{n\times d_{c}}$, the local embedding is $w_{l}=s_{n}$, and the global embedding is computed as

(7) $$\begin{array}{c} \alpha_{i}=q^{⊺}\sigma (W_{1}w_{l}+W_{2}s_{i}+b)\\ w_{g}=\alpha \cdot s \end{array}$$

with $q\in R^{d_{c}}$, $W_{1},W_{2}\in R^{d_{c}\times d_{c}}$, and bias *b* being learnable parameters. To improve the chance of a tool being recommended that conforms with recent context, the latest tool in the sequence is emphasized by concatenating the global workflow embedding $w_{g}$ with the local workflow embedding $w_{l}$. The final representation is obtained by compressing this concatenation with a learned matrix $W_{3}\in R^{d_{c}\times 2d_{c}}$ and further to the space of the tool ID embedding ($R^{d_{t}}$) using matrix $W_{4}\in R^{d_{t}\times d_{c}}$.

(8) $$w_{c}=W_{3}[w_{l}∥w_{g}].$$

(9) $$w_{s}=W_{4}w_{c}.$$

We introduce two matrices, $W_{3}$ and $W_{4}$, as distinct entities. In a prior experiment iteration, we combined $W_{3}$ and $W_{4}$ into a single matrix in Equation (8) to transition from $2d_{c}$ to $d_{t}$. However, we observed slightly inferior results with this approach. Furthermore, maintaining $W_{3}$ and $W_{4}$ as separate matrices emphasizes that the dimensionality of an embedded workflow ($d_{c}$), does not have to align with the size of a tool embedding.

### 2.5 Tool recommendation

With the final embedded representation of a workflow-in-progress obtained, recommended tools are calculated. Each tool in the dataset is ranked based on the degree of positional similarity between its embeddings and the final workflow embedding $w_{s}$. This is calculated simultaneously by multiplying the sequence embedding with the learned tool embedding weights matrix $W_{\mathrm{tool}}$ of Equation (1) as follows

(10) $$\hat{y}=g({\left(W_{\text{tool}}\times w_{s}^{⊺}\right)}^{⊺}),$$

where *g* is a function like softmax for probabilities. $\hat{y}\in R^{u}$ is the vector of probabilities of each tool appearing next in the workflow.

When multiple tools suit the same task, BTR recommends and ranks them. The current implementation displays the top-*n* tool suggestions for a given query, with *n* set to 5 by default (configurable). The dynamic choice of *n* is possible by considering score thresholds assigned to each tool. During the ranking of top-*n* tools, if they apply to the same task, the model uses its weightings based on contextual matching, influenced by tool usage frequency in the training data.

### 2.6 Model implementation

The models are implemented in Python using PyTorch (Paszke *et al.* 2019) and PyTorch Geometric (Fey and Lenssen 2019) libraries. Each model trains to reduce the cross entropy loss between the prediction and ground truth of every workflow query, defined as

(11) $$\text{LCE}(\hat{y})=-\frac{1}{u}\sum_{i=1}^{u}y_{i}\cdot \;\text{log}({\hat{y}}_{i})+(1-y_{i})\cdot \;\text{log}(1-{\hat{y}}_{i}),$$

where $\hat{y}$ are the tool probabilities from Equation (10), $y\in R^{u}$ is a one-hot encoded vector of the ground truth. Back-propagation through time is used to compute the gradients (Mozer 1995). Dropout is applied to initial tool embeddings and to the aggregated node embeddings after GG-NN layer. Mini-batch Adam optimizer (Kingma and Ba 2015) is employed to update the weights. Hyperparameters include learning rate, how much it decays by and how often, L2 penalty, batch size, number of epochs, and dropout rates. These are initialized with ranges and Bayesian optimization is used to select the values. We optimize hyperparameters over 10 training iterations and use those values for training the model.

### 2.7 Datasets and training

The proposed method is evaluated using two datasets, both which are collections of previously-created Galaxy (Afgan *et al.* 2018) workflows. **AllGalaxy:** The first dataset, dubbed AllGalaxy, is the set of all workflows that are publicly available for viewing and download from the three UseGalaxy servers: usegalaxy.org, usegalaxy.eu, and usegalaxy.org.au (Afgan *et al.* 2018, The Galaxy Community 2022, Afgan *et al.* 2015). Table 1 contains information about the workflow data.

**Table 1.** Information regarding the datasets collected for evaluation.

Data source	Workflows	Tools	Max steps	Avg steps	Med steps
usegalaxy.org	892	564	142	16.8	11
usegalaxy.eu	813	1004	145	15.1	10
usegalaxy.org.au	321	504	71	17.3	13
Combined	2026	1295	145	16.2	11
AllGalaxy^a^	1367	1276	47	11.8	9
EuGalaxy^a^	5782	1359	50	9.9	7

a Indicates the data has been filtered to unique workflows of length 2–50.

**EuGalaxy:** The second dataset, EuGalaxy, is provided by Kumar *et al.* (2021) alongside their proposed Galaxy tool recommendation method. This dataset consists of public and private, potentially invalid or deleted workflows from the usegalaxy.eu server. We use a snapshot of this dataset from April 2020 provided on their GitHub repository (https://github.com/anuprulez/galaxy_tool_recommendation) and remove workflows labeled as erroneous.

Before training, the datasets are filtered to prevent bias and overfitting. Workflows exceeding 50 steps are discarded due to their rare occurrences. Workflows with identical tool invocations are de-duplicated to retain one instance. The filtered datasets are divided into 80% train, 10% validation, and 10% test sets, which are observed to be common split sizes in machine learning. The division occurs over the set of workflows so that individual workflows are fully contained within their respective set. Each full workflow is then iteratively split into prefix-graphs or prefix-sequences from start to finish so the model can perform tool recommendation at any stage of workflow development. These become the final queries used for training/testing. An ensemble of 20 evaluation models per variant are trained over different random train/test/val splits and the results are averaged. Optimizing hyperparameters and training all 20 evaluation models takes no longer than eight hours for the slowest variant using an RTX 3060 laptop GPU. Models are <30 MB on disk, scaling with toolbox size rather than number of workflows or training queries.

### 2.8 Evaluation details

The architecture is evaluated by training several variants with different experimental configurations and comparing the performance with metrics that aim to capture the system’s utility for workflow construction. The metrics measure recommendation accuracy, whereby a recommendation is considered correct if it matches the single ground truth tool for each query, as opposed to a list of potential relevant items that other recommendation systems may use. From this the following three metrics are used.

- **HR@1**: The rate at which the very first tool the model recommends matches the ground truth.
- **HR@3**: The rate at which any of the first three recommended tools match the ground truth.
- **MRR@5**: Mean Reciprocal Rank is a positional-aware measure of recommendation quality that penalizes the ground truth item appearing lower in the recommendation list. This is an appropriate metric as the model should recommend the correct item earlier to save time for a user inspecting the recommendations. It gives an idea of the model’s ability to highly-rank the correct tool.
  $$\begin{array}{c} \text{MRR}@5=\{\begin{array}{cc} \frac{1}{i}, & \text{if}\;i\leq 5\\ \;0, & \text{otherwise} \end{array} \end{array},$$

where *i* is the 1-based index of the correct tool in the ranked tool list.

## 3 Results

### 3.1 Experiments conducted

Variants of ${\text{BTR}}^{g}$, over graphs, and ${\text{BTR}}^{s}$, over linear sequences, are trained and evaluated with the automated metrics from Section 2.8. We are interested in determining the impact of architecture components, so ablation studies are performed with models that build up to the full architecture. All models are prepared in the same manner as Section 2.6. The first models presented, ${\text{BTR}}_{\text{ATTN}}^{*}$, include the attention mechanism that aggregates tool embeddings into a latent workflow vector. They do not incorporate PubMedBERT description vectors and therefore only need toolbox ID as an input feature. The next models are ${\text{BTR}}_{\text{NLP}}^{*}$, which include PubMedBERT description vectors as described in Section 2.4.1. These models do not use an attention mechanism and instead use mean-pooling to calculate the workflow node activations. The third models are ${\text{BTR}}_{\text{ATTN}+\text{NLP}}^{*}$ and incorporate the full BTR architecture as previously described.

BTR is evaluated against the only closely related work by Kumar *et al.* (2021), the GTR of the usegalaxy.eu server. The pre-trained model (over EuGalaxy) is obtained from the author’s GitHub repository and evaluated with the same metrics over the same test data. The pre-trained GTR model gives two sets of recommendations—one for what is defined as the high-quality, shared workflows, and another for unshared workflows. We discard the distinction between shared and unshared, the evaluation metrics are calculated for each of these sets and the higher value is taken per input to remain fair. GTR is not evaluated against AllGalaxy as we cannot obtain the unshared workflows for all usegalaxy servers that are needed to train the model.

### 3.2 Experimental findings

Results are summarized in Table 2. The key findings for the three main experiments are as follows.

**Table 2.** Performance of BTR models using evaluation metrics from Section 2.8, compared to the baseline Galaxy tool recommendation method (GTR).

Model	HR@1	AllGalaxy HR@3	MRR@5	HR@1	EuGalaxy HR@3	MRR@5
Baseline (Kumar *et al.* 2021)				31.22%±2.69%	60.46%±3.38%	46.64%±2.76%
${\text{BTR}}_{\text{ATTN}}^{g}$	40.70%±4.19%	59.04%±3.86%	50.39%±3.96%	45.59%±1.42%	67.66%±1.46%	57.19%±1.32%
${\text{BTR}}_{\text{NLP}}^{g}$	41.42%±4.44%	59.33%±4.84%	51.08%±4.20%	48.22%±1.42%	70.23%±1.40%	59.72%±1.33%
${\text{BTR}}_{\text{ATTN}+\text{NLP}}^{g}$	**44.15%** ± **3.78%**	**60.54%** ± **3.89%**	**52.84%** ± **3.68%**	**51.21%** ± **1.33%**	**71.36%** ± **1.32%**	**61.68%** ± **1.21%**
${\text{BTR}}_{\text{ATTN}}^{s}$	31.98%±3.51%	54.28%±4.52%	43.90%±3.79%	38.30%±2.55%	66.30%±3.66%	52.93%±3.15%
${\text{BTR}}_{\text{NLP}}^{s}$	**34.43%** ± **4.12%**	53.55%±5.06%	44.24%±4.28%	44.04%±2.19%	73.25%±3.05%	59.23%±2.60%
${\text{BTR}}_{\text{ATTN}+\text{NLP}}^{s}$	33.72%±4.36%	54.27%±4.78%	44.45%±4.41%	**47.16%** ± **2.13%**	**75.13%** ± **3.20%**	**61.49%** ± **2.50%**

The values are the mean and standard deviation of the metrics from 20 evaluation models using random data splits.

Values in bold represent the clear-cut top score within the category.

#### 3.2.1 Graph representations improve performance

**Figure 3.**
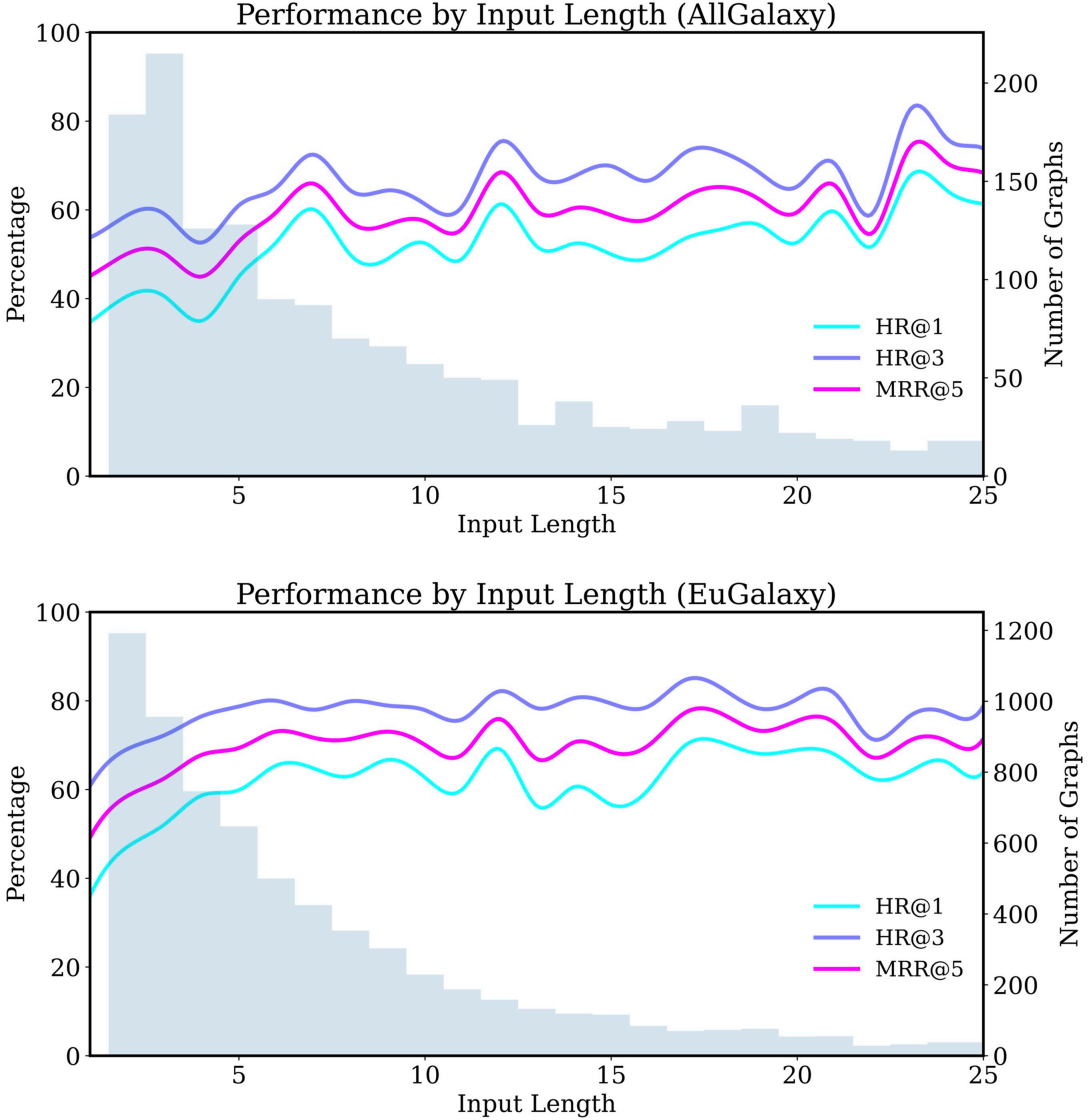
Performance of ${\text{BTR}}^{g}$ by the input query length. The lines represent the average of the metrics from Section 2.8 at each input length and are shown as percentages matching Table 2. The bars and right-side axis show the length distribution of workflow graphs in the datasets and are included to explain noise seen in the plots.

${\text{BTR}}^{g}$ using graph representations for workflows-in-progress outperforms ${\text{BTR}}^{s}$ using linear tool sequences. Direct, assured comparison between graph and sequence cannot be made because the data structures and training splits are different. ${\text{BTR}}^{g}$ represents the full preceding context within a query, so there is only one query per ground-truth node in the workflows. ${\text{BTR}}^{s}$ can have multiple queries extracted where recent tool sequences are identical, but diverge earlier upstream. Nevertheless, we perceive the performance as coverage over the datasets, for which ${\text{BTR}}^{g}$ excels. Note the significant gap in metrics between the AllGalaxy-trained models. This suggests that the graph representation can yield strongly preferable models when less data is available. ${\text{BTR}}^{g}$ displays improved stability across evaluation models, implying the graph representation is less sensitive to differences in data splits. The mean percentage for the metrics in the table do not show a complete picture of model performance. Figure 3 is included to visualize the evaluated metrics with different input lengths. As expected, it is observed the metrics generally increase as input length increases, with reduced effects after around length 8. The noise at larger lengths is attributed to lower numbers of available testing queries.

#### 3.2.2 Attention and NLP are impactful

The results show that both NLP and attention have notable impacts on model performance. Notice is drawn towards AllGalaxy’s ${\text{BTR}}_{*}^{s}$ variations, which do not show as clear of a trend. We speculate that as the worst performing models, using small amounts of high-variability sequential data, it is unable to make good use of the semantic features this component provides. For the rest of the models, we observe that including short descriptions has high impact indicated by the lacking variation’s (${\text{BTR}}_{\text{ATTN}}^{*}$) degraded performance. The attention mechanism does not have as substantial of an effect, but still a noteworthy improvement is seen from ${\text{BTR}}_{\text{NLP}}^{*}$ to ${\text{BTR}}_{\text{ATTN}+\text{NLP}}^{*}$. We conclude that both components are important features and in general improve the model performance.

#### 3.2.3 BTR significantly outperforms the baseline system, GTR

BTR shows a 50% improvement over the GTR, the baseline model, in recommending the correct ground-truth tool for a given query tool sequence, as measured by HR@1. The closest comparison to GTR from an architecture standpoint is with ${\text{BTR}}^{s}$. This is because both models use linear tool sequences to represent queries and are evaluated on the exact same data and representation (comma-separated tool sequence). ${\text{BTR}}_{\text{ATTN}+\text{NLP}}^{s}$ demonstrates consistent performance gain of +15% across all categories in EuGalaxy data. Note that GTR cannot run the AllGalaxy data because the unshared workflow data is not available for training. This finding underlines the potential utility of BTR during workflow construction. Furthermore, we reassert that ${\text{BTR}}^{g}$ gives better coverage of the EuGalaxy data and an implemented system should use it to leverage the graph representation.

### 3.3 Case studies of full tool recommendation

**Figure 4.**
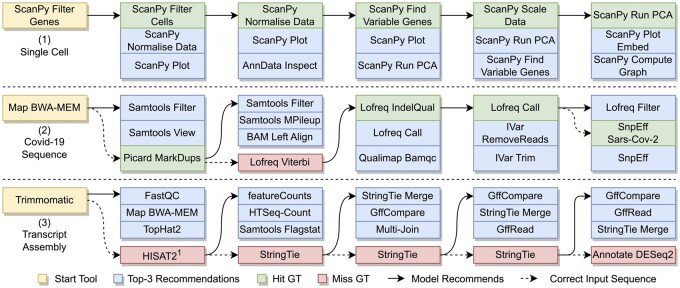
Three examples of sequential workflow tool recommendations using an AllGalaxy-trained ${\text{BTR}}_{\text{ATTN}+\text{NLP}}^{s}$ model. At each step, the correct recommendation or ground truth is included as part of the query for the next step. Examples (1) and (2) show robust utility of automated tool recommendation; Example (3) showcases highly customized workflow where recommendation accuracy can only be improved with additional user input. In cases when the recommended tools do not match ground truth, we observe that they are generally relevant and can be appropriate in different use cases. HISAT2, denoted with ^1^, is the fourth ranked tool recommendation for the input.

Figure 4 shows three examples where a series of tool recommendations are conducted as a sequence, including workflows for (i) Single cell analysis, (ii) COVID-19 Variation analysis, and (iii) transcript assembly. The Single cell workflow demonstrates the model’s ability to chain together full workflow sequences given a starting tool. The COVID-19 workflow sees an instance where user intent could not be determined, but corrects the sequence from thereafter. The transcript assembly workflow shows a highly specific use case where the model cannot capture the user’s intentions without additional input. Note that the recommendations provided by our model are highly relevant nonetheless. This highlights a limitation of the model that is discussed later on.

### 3.4 BTR comparing to large language models

AI chatbots such as ChatGPT, powered by large language models like GPT-3.5 and GPT-4 (OpenAI 2022), are up-and-coming technologies that have the potential to be disruptive. Scientists from diverse disciplines have begun to investigate how the technology can be leveraged, including applications to Bioinformatics. Shue *et al.* (2023) provide ways ChatGPT can be used by students for solving problems and resolving errors. They find the chatbot demonstrates promising utility, however when presented with complex tasks it can start to hallucinate. In Lubiana *et al.* (2023), tips from different categories are given to describe ways this technology may enhance the routine work of Bioinformatics researchers.

We are interested in exploring how BTR stacks up with the capabilities of ChatGPT. A brief study is conducted to provide some outlook. 100 sequence queries of length 3–10 are randomly selected from the test set of an EuGalaxy ${\text{BTR}}^{s}$ evaluation model. The top-3 recommendations are obtained from the model and from ChatGPT, which is constrained to the input/output format of BTR. Few-shot prompting is used to obtain the ChatGPT recommendations. Three examples of inputs and corresponding top-3 recommendations are provided, the chatbot is asked to give the top-3 tools for the new sequence in the same manner. The prompt used and 100 sample results are available as Supplementary Material.

The metrics for the sequences are calculated and displayed in Table 3. From the metrics alone, it appears that the general chatbot cannot perform as well as a specialized system for direct Galaxy tool recommendation. We imagine a major reason being ChatGPT is not fine-tuned for the Galaxy tool recommendation task; though it did have the potential to train on all of the tools and workflows present. In general, ChatGPT often fails to provide the desired functionality based on the workflow-in-progress. When given the correct tool for a sequence and asked why it could not provide it, the chatbot responded “… I’m not aware of the specific context or requirements of your analysis, and my response was based solely on the tool sequence you provided…” The chatbot needs more information to provide correct recommendations, which is not required by BTR.

**Table 3.** Performance of ChatGPT over 100 random samples from EuGalaxy.

Model	HR@1	HR@3
ChatGPT	5%	10%
${\text{BTR}}_{\text{ATTN}+\text{NLP}}^{s}$	55%	78%

Table 4 includes examples chosen to highlight the behaviors of the two methods. Each row of the table contains a Sample ID corresponding to a row in the full results from Supplementary Material. Sample 11 demonstrates a positive result where ChatGPT ranks the correct tool in the first position. Sample 3 shows an incorrect recommendation in which BTR succeeds. Sample 31 shows ChatGPT recommending an outdated implementation of a functionality. The new tool description notes it is rewritten in modern Python, leading one to believe it is a better choice to use. The new version came out before ChatGPT’s training cut-off. In sample 56 we observe the chatbot’s tendency to hallucinate and make up tools/functionalities. The first recommended tool does not actually exist, which could mislead a user. Other cases of hallucination include ChatGPT recommending correct functionality but a partially invalid tool ID (join1 - > join2). We consider this a correct recommendation, though automated toolbox retrieval may fail in this instance. Samples 97 and 85 denote examples where ChatGPT fails to provide any recommendations. In the first case, it erroneously claims that the input tools do not exist within Galaxy. In the second, the first input tool is custom-uploaded, though the rest are available.

**Table 4.** Examples of BTR recommendations alongside few-shot-prompting of ChatGPT to highlight different behaviors.

ID	Sequence	Ground-Truth	BTR Top-3	ChatGPT Top-3	Comments
11	[…] gatk2_print_reads, gatk2_reduce_reads, gatk2_haplotype_caller	gatk2_variant _recalibrator	gatk2_variant_select, gatk2_variant_apply_ recalibration, gatk2_variant_recali-brator	gatk2_variant_recali-brator, gatk2_variant_filtra-tion, snpeff_annotate	Both BTR and Chat-GPT are correct
3	bowtie2, freebayes, SnpEff-cds-report, gemini_load	gemini_db_ info	gemini_db_info, gemini_query, snpEff	gemini_annotate, gemini_query, gemini_stats	BTR is correct while Chat-GPT is incorrect
31	fastq_groomer, bowtie2, deeptools_compute-Matrix	deeptools_ heatmapper	deeptools_ heatmapper, deeptools_plot_profile, deeptools_profiler	bamCoverage, computeGCBias, plotHeatmap	BTR is correct, Chat-GPT is incorrect but provides an outdated version of correct tool
56	[…] tp_replace_in_column, CONVERTER_interval_ to_bed_0, gops_intersect_1	datamash_ops	Grouping1, datamash_ops, Count1	CONVERTER_bed_ to_interval_0, BEDTools_merge, BEDTools_sort	BTR is correct. Chat-GPT is incorrect and hallucinates a tool name that does not exist
97	gspan, nspdk_sparse, NSPDK_candidateClust	NSPDK_can-didateClust	preMloc, NSPDK_candidate-Clust, tp_awk_tool	NA	BTR is correct, Chat-GPT fails to provide a recommendation
85	secretbt2test, bam_to_sam, tp_awk_tool, extract_aln_ends.py, merge_pcr_duplicates.py	rm_spurious_ events.py	rm_spurious_events.py, mothur_summary_ single, mothur_venn	NA	BTR is correct, Chat-GPT fails to provide a recommendation because it does not recognize the first tool of input sequence

The IDs match the index of the sample in the complete set. The tools shown are the tool identifiers within Galaxy, which the models take as input. A highlighted name in the Top-3 indicates a correct recommendation. Underlined tools are referred to in the comments.

## 4 Discussion

Developing Bioinformatics workflows is demanding, involving critical considerations, especially with the complexity of a vast tool catalog. We introduce BTR, a novel approach to Bioinformatics workflow tool recommendation. Framing workflow construction as a session-recommendation problem, we use graph neural networks to capture the workflow in a graph representation. Embeddings from a language model enhance recommendation quality, with an attention mechanism focusing on relevant workflow history. Experiments show BTR outperforms an existing Galaxy system, and in a brief study, it surpasses ChatGPT in recommending Galaxy tools based on workflow step sequences.

We envision the BTR architecture for workflow recommendation can be implemented as a standalone application or be incorporated into a plug-in for existing workflow management systems such as Galaxy. Its utility can be further augmented by including additional information following each recommendation, such as links to the user manual, sample codes, and suggested parameterizations. We believe such system will provide instant guidance for Bioinformatics developers during the construction of workflows in an unfamiliar domain, significantly shortening development time needed. Additionally, the system has potential to enhance the quality of constructed workflows by learning from completed processes, effectively sidestepping mistakes, and pitfalls.

The proposed system can be readily extended to incorporate tool parameters and configuration options. These options can be important context for tool use, as the function of some tools may change considerably with different configurations. Furthermore, configuring and optimizing the selected tools is another challenging and time-consuming task of workflow construction. Instances of a specific tool may have shared or overlapping configurations that can be matched throughout the workflows. If multiple sets of configurations with several instances each are obtained, parameter information can be incorporated into BTR. This is accomplished by extracting these configuration sets as separate tools in the toolbox. Descriptions for the different configurations could be appended with annotation data from the workflow files to further improve the recommendation quality.

Nevertheless, we foresee several actionable areas of improvements that can further increase the accuracy and utility of BTR in the next stage of its development. Further information about tools such as supported hardware, popularity, functional category, and input/output types can be incorporated into the architecture as additional node or edge features. One limiting factor of the model performance is the amount of workflow data to train on. We speculate that the performance would continue to improve with diminishing returns as the size and generality of datasets increase. The graph representation (AWR) adopted by our model can be extended by including other workflow databases or sources including Snakemake (Mölder *et al.* 2021) and Common Workflow Language (Crusoe *et al.* 2022), both of which consist of workflow files that can be parsed into a DAG of labeled steps. The challenge with utilizing these different workflows is that, unlike Galaxy, the frameworks often do not pull from a consistent repository of tools. Consideration is needed to match tools between workflows that may have inconsistent annotations, and to further obtain corresponding tool descriptions.

Expanding user control during the recommendation process is another area for improvement. Users of the system have limited ways to control recommendations aside from the set of nodes selected to lead into the recommended tool. In the case of Fig. 4(3), additional user input at each stage such as “transcript assembly” may lead to correct recommendation. We believe the NLP component enables a modified version of the model to be prompted with natural language descriptions of the desired functionality or goal to appear next. Abstract keywords capturing intent may be used to direct the recommendations. One way to experiment with this is to shift the description embeddings to the prior step in the queries, pooling them for nodes with multiple outgoing edges. Into the last node(s), the embedded user-input text would be placed. Another option is to add the text as the description of the query node. Careful consideration needs to be taken when training and evaluating such a model, as the ground truth may be part of the input in some way.

## Supplementary Material

btae275_Supplementary_Data

## Data Availability

Instructions for obtaining and processing the datasets used to evaluate the models are available at https://github.com/ryangreenj/bioinformatics_ tool_recommendation.
